# Glucagon-like-peptide-1 receptor agonists in the intensive care unit: practical implications for the intensivists

**DOI:** 10.62675/2965-2774.20260166

**Published:** 2026-08-06

**Authors:** Larissa Bianchini, Gloria Adriana Rocha Martins, Sheila Nainan Myatra

**Affiliations:** 1 HCor-Hospital do Coração Research Institute São Paulo SP Brazil Research Institute, HCor-Hospital do Coração - São Paulo (SP), Brazil.; 2 Instituto D’Or de Pesquisa e Ensino Department of Critical Care and Postgraduate Program Rio de Janeiro RJ Brazil Department of Critical Care and Postgraduate Program, Instituto D’Or de Pesquisa e Ensino - Rio de Janeiro (RJ), Brazil.; 3 Homi Bhabha National Instiutute Department of Anaesthesiology, Critical Care and Pain, Tata Memorial Hospital Mumbai India Department of Anaesthesiology, Critical Care and Pain, Tata Memorial Hospital, Homi Bhabha National Instiutute - Mumbai, India.

The use of glucagon-like peptide-1 receptor agonists (GLP-1 RAs) has increased in recent years, with uptake reaching 6% of the general population in the United States and exceeding 20% among patients with diabetes.^(1)^ Beyond improving glycemic control, these agents reduce major adverse cardiovascular^(2)^ and renal morbidity and are widely prescribed for weight management. Glucagon-like peptide-1 receptor agonists delay gastric emptying, with reported increases in regurgitation and pulmonary aspiration during airway management under anesthesia, and complicate glycemic control in the critically ill.^(3)^ As intensivists encounter patients on these therapies with increasing frequency, understanding their pharmacologic effects is essential for optimizing care in the intensive care unit (ICU) (Figure 1).

**Figure 1:**
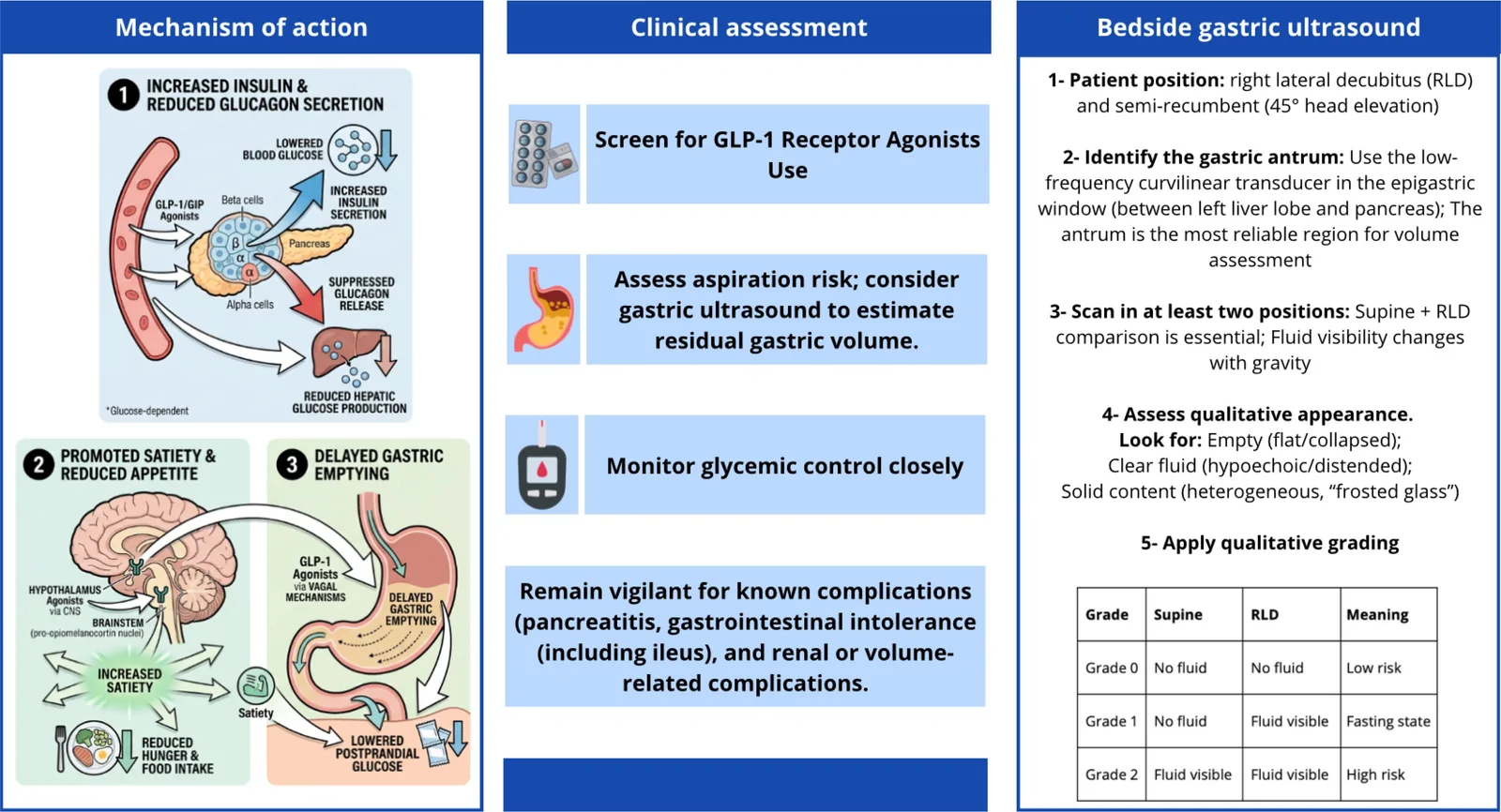
Overview of glucagon-like peptide-1; glucose-dependent insulinotropic polypeptide receptor agonists in critical care: mechanisms of action, a stepwise clinical assessment approach, and key elements of bedside gastric ultrasound for aspiration risk evaluation.^(4)^

GLP-1 glucagon-like peptide-1; GIP - glucose-dependent insulinotropic polypeptide; CNS – central nervous system; RLD – right lateral decubitus.

Figure partially generated with Artificial Intelligence-assisted tools. The authors reviewed and validated all content.

## GLUCAGON-LIKE PEPTIDE-1 PHYSIOLOGY

The GLP-1 hormone is released from small intestinal cells in response to food intake and increases insulin secretion through a glucose-dependent mechanism. Endogenous GLP-1 levels rise during the early phase of sepsis as compensation for stress hyperglycemia and systemic inflammation, with levels tracking pro-inflammatory mediators, including interleukin-6 (IL-6), tumor necrosis factor-α (TNF-α), and lipopolysaccharide (LPS)-related signaling. Elevated GLP-1 concentrations are associated with early death and prolonged critical illness, marking dysregulation of the incretin system and disease severity.^(5)^

## GLUCAGON-LIKE PEPTIDE-1 RECEPTOR AGONISTS

Glucagon-like peptide-1 receptor agonists mimic the endogenous hormone and exert their glucose-lowering effects through three main mechanisms. First, they act on glucose-dependent insulin secretion by pancreatic beta cells and suppress glucagon release by alpha cells, reducing hepatic glucose production. Second, they promote satiety via central nervous system pathways involving the hypothalamus and brainstem. Third, they delay gastric emptying through vagal-mediated mechanisms. Approved agents include exenatide, liraglutide, dulaglutide, and semaglutide, while tirzepatide acts as a dual GIP/GLP-1 RA and produces greater weight loss compared to selective GLP-1 RAs. Half-lives range from approximately 1.4 hour for exenatide to 13 - 15 hours for liraglutide, extending to nearly 120 hours for tirzepatide and up to 180 hours for semaglutide. Tachyphylaxis to the gastric emptying effect may develop with prolonged use of long-acting agents.^(3)^

## GLUCAGON-LIKE PEPTIDE-1 RECEPTOR AGONISTS AND CRITICAL ILLNESS

Hyperglycemia in the critically ill arises from multifactorial mechanisms, driven by insulin resistance and excess glucagon secretion. Small studies investigated intravenous GLP-1 amide as an adjunct to insulin for glycemic stabilization, without increased risk of hypoglycemia.^(6,7)^ These formulations are not commercially available, even though they appear to have a favorable side-effect profile. Liraglutide, when compared to intravenous insulin in critically ill patients, achieved similar glycemic targets, though at the cost of greater gastrointestinal adverse effects, mainly nausea.^(8)^

Preclinical evidence suggests that GLP-1 RAs attenuate muscle catabolism by suppressing atrophic pathways and promoting myogenic signaling.^(9)^ A potential role in acute respiratory distress syndrome through reduced inflammation, alveolar-capillary barrier protection, and decreased cytokine production is also under study.^(3)^

## EVIDENCE FROM PERI-PROCEDURE PRACTICE AND CRITICAL CARE

As GLP-1 RAs retard gastric emptying by vagal and enteric mechanisms, patients receiving long-acting agents may present with residual gastric volume despite adherence to standard fasting recommendations. This delay increases the risk of regurgitation and pulmonary aspiration during procedural sedation and emergency intubation.

The American Society of Anesthesiologists (ASA) addressed these concerns in recent guidance, recommending that daily formulations be withheld on the day of elective procedures and that weekly formulations be withheld for up to 7 days prior to anesthesia.^(10)^ Fasting from solids 24 hours before procedures requiring anesthesia and liquid fasting for 4 - 8 hours, depending on its caloric content, is recommended regardless of having stopped the medication.^(11)^ In critically ill patients, this risk becomes amplified: urgent airway interventions frequently occur without fasting or drug washout, and baseline gastrointestinal dysmotility, related to opioids, vasopressors, and systemic inflammation, magnifies the problem. Recent GLP-1 RA exposure warrants active assessment during the pre-intubation evaluation. A recent multidisciplinary consensus statement^(12)^ on the perioperative management of adults taking GLP-1 RAs recommends using point-of-care gastric ultrasound to stratify pulmonary aspiration risk prior to induction of anesthesia. Measures to reduce the risk of pulmonary aspiration include administering prokinetics, using a modified rapid sequence intubation, maintaining a head-up position, and considering awake intubation.

From a nutritional standpoint, delayed gastric emptying may justify early post-pyloric feeding access in those with poor gastric tolerance.^(3)^ Evidence specific to critically ill populations remains scarce, with most recommendations extrapolated from perioperative data. Prospective studies evaluating gastric motility, aspiration risk, and enteral feeding outcomes among ICU patients exposed to GLP-1 RAs are urgently needed. Until such data emerge, individualized risk assessment and heightened clinical vigilance remain the standard of care.

## MAJOR COMPLICATIONS

Although generally well tolerated, GLP-1 RAs entail relevant complications that may precipitate hospitalization or complicate the management of critically ill patients.

## PANCREATITIS AND BILIARY DISEASE

Acute pancreatitis is reported with GLP-1 RAs, with uncertain causality. Albeit large outcome trials indicate a low absolute risk, severe cases progressing to systemic inflammatory response syndrome, organ dysfunction, and hemodynamic instability necessitating ICU-level support were described.^(3,13)^ Emerging evidence suggests the pancreatic signal reflects an indirect biliary phenomenon, mediated by rapid weight loss and gallbladder dysmotility, rather than direct pancreatic toxicity. Consistent with this mechanism, GLP-1 RAs associate with a 37% increased risk of biliary complications, including cholelithiasis, cholecystitis, and cholecystectomy, with risk appearing dose- and duration-dependent.^(14)^

## GASTROINTESTINAL INTOLERANCE AND ILEUS

As previously described, GLP-1 RAs delay gastric emptying and intestinal motility, which in hospitalized patients exacerbates nausea, vomiting, ileus, and feeding intolerance.^(3)^

## RENAL AND VOLUME COMPLICATIONS

Renal dysfunction associated with GLP-1 RAs typically results from a prerenal mechanism related to gastrointestinal losses, independent of direct nephrotoxicity. Pharmacovigilance data identified 2,670 cases of GLP-1 RA-associated acute kidney injury, with a median onset of 63 days, a hospitalization rate of 45%, and mortality reaching 4.23%, with obese and elderly patients at greatest risk.^(15)^

## CONCLUSION

Glucagon-like peptide-1 receptor agonists present both intended and undesirable effects with direct relevance to critical care. Active assessment of recent exposure informs safer airway strategies, optimizes nutritional support, and guides metabolic management. Despite remaining uncertainties, understanding their pharmacologic profile and potential complications is essential for appropriate, individualized patient care.

## Data Availability

The contents underlying the research text are included in the manuscript.
